# Involvement of TGF-β1/Smad3 Signaling in Carbon Tetrachloride-Induced Acute Liver Injury in Mice

**DOI:** 10.1371/journal.pone.0156090

**Published:** 2016-05-25

**Authors:** Liman Niu, Xueling Cui, Yan Qi, Dongxue Xie, Qian Wu, Xinxin Chen, Jingyan Ge, Zhonghui Liu

**Affiliations:** 1 Department of Immunology, College of Basic Medical Sciences, Jilin University, Changchun, China; 2 Department of Genetics, College of Basic Medical Sciences, Jilin University, Changchun, China; 3 Department of Physiology, College of Basic Medical Sciences, Jilin University, Changchun, China; National Institutes of Health, UNITED STATES

## Abstract

Transforming growth factor-beta1 (TGF-β1) is a major factor in pathogenesis of chronic hepatic injury. Carbon tetrachloride (CCl_4_) is a liver toxicant, and CCl_4_-induced liver injury in mouse is a classical animal model of chemical liver injury. However, it is still unclear whether TGF-β1 is involved in the process of CCl_4_-induced acute chemical liver injury. The present study aimed to evaluate the role of TGF-β1 and its signaling molecule Smad3 in the acute liver injury induce by CCl_4_. The results showed that CCl_4_ induced acute liver injury in mice effectively confirmed by H&E staining of liver tissues, and levels of not only liver injury markers serum ALT and AST, but also serum TGF-β1 were elevated significantly in CCl_4_-treated mice, compared with the control mice treated with olive oil. Our data further revealed that TGF-β1 levels in hepatic tissue homogenate increased significantly, and type II receptor of *TGF-β (TβRII)* and signaling molecules *Smad2*, *3*, mRNA expressions and Smad3 and phospho-Smad3 protein levels also increased obviously in livers of CCl_4_-treated mice. To clarify the effect of the elevated TGF-β1/Smad3 signaling on CCl_4_-induced acute liver injury, Smad3 in mouse liver was overexpressed *in vivo* by tail vein injection of Smad3-expressing plasmids. Upon CCl_4_ treatment, Smad3-overexpressing mice showed more severe liver injury identified by H&E staining of liver tissues and higher serum ALT and AST levels. Simultaneously, we found that Smad3-overexpressing mice treated with CCl_4_ showed more macrophages and neutrophils infiltration in liver and inflammatory cytokines IL-1β and IL-6 levels increment in serum when compared with those in control mice treated with CCl_4_. Moreover, the results showed that the apoptosis of hepatocytes increased significantly, and apoptosis-associated proteins Bax, cytochrome C and the cleaved caspase 3 expressions were up-regulated in CCl_4_-treated Smad3-overexpressing mice as well. These results suggested that TGF-β1/Smad3 signaling was activated during CCl_4_-induced acute liver injury in mice, and Smad3 overexpression aggravated acute liver injury by promoting inflammatory cells infiltration, inflammatory cytokines release and hepatocytes apoptosis. In conclusion, the activation of TGF-β signaling contributes to the CCl_4_-induced acute liver injury. Thus, TGF-β1/Smad3 may serve as a potential target for acute liver injury therapy.

## Introduction

Transforming growth factor-beta (TGF-β) superfamily, which includes TGF-β, inhibins, activin and bone morphogenetic protein (BMP), possesses a wide range of biological function. They play critical roles in early embryogenesis, neuronal differentiation induction, hematopoiesis and osteoblast proliferation and differentiation [1–4]. Numerous studies have shown that abnormal expression of TGF-β1 is involved in the process of virus hepatitis, hepatic fibrosis, liver cancer, hepatic failure and other chronic hepatic diseases [5–9], and TGF-β1 exerts important roles in inhibition of hepatocyte proliferation and promotion of extracellular matrix (ECM) production [10–12]. Consequently, TGF-β1 has been considered as a key molecule in the formation of hepatic fibrosis and pulmonary fibrosis [13]. TGF-β receptors are serine/threonine kinase receptors. In general, signaling is initiated with TGF-β/TGF-β type II receptor (TβRII) binding and subsequently, type I receptor (TβRI) is activated. The activated TβRI phosphorylates and activates Smads, which is a family of the drosophila gene mothers against decapentaplegic (Mad) homologs. Smad3 is an important TGF-β signaling molecule [14] and exerts important roles in activation of hepatic stellate cells, specifically inducing them to produce concomitant extracellular matrix [15, 16].

Liver is an important metabolic organ in the body, and can be injured by various factors, including trauma, viral infection, chemical reagents, and so on [17, 18]. Carbon tetrachloride (CCl_4_) is commonly accepted as a typical liver toxicant, and CCl_4_-induced liver injury is a classic model of chemical liver injury in mice [19]. It was reported that TGF-β1 protein level was elevated significantly in CCl_4_-induced chronic liver injury in mice [10, 20], and recent study has revealed that Smad3 mRNA expression increased in CCl_4_-induced acute liver injury in mice [17]. However, the TGF-β1 protein expression and the effect of TGF-β1/Smad3 signaling on CCl_4_-induced acute liver injury are still unclear. Therefore, in the present study, the level of TGF-β1 protein in serum and hepatic tissue homogenate of CCl_4_-induced acute liver injury mice were first determined. Furthermore, we overexpressed the TGF-β signaling molecule Smad3 in mouse liver *in vivo* to define the effect of TGF-β1/Smad3 signaling on CCl_4_-induced acute liver injury in mice.

## Materials and Methods

### Reagents

CCl_4_ was purchased from Shantou West Long Chemical Co., Ltd (Shantou, China). Enzyme-linked immunosorbent assay (ELISA) kits for TGF-β1, IL-1β and IL-6 were purchased from BD Biosciences (San Diego, USA). Rabbit anti-mouse Smad3 and anti-Bax polyclonal antibodies were purchased from ABclonal Biotech Co., Ltd (Cambridge, USA), anti-cleaved caspase 3 antibody from Cell Signaling Technology, Inc (Boston, USA), anti-GAPDH and anti-phospho-Smad3 polyclonal antibodies from Sungene Biotech Co., Ltd (Tianjin, China), and mouse anti-Bcl-2 and cytochrome C (Cyt C) monoclonal antibodies from BD Biosciences (San Diego, USA). Terminal UTP nick-end labeling (TUNEL) staining kit was purchased from Roche (Mannheim, Germany). Alanine aminotransferase (ALT) and aspartate aminotransferase (AST) assay kits were provided by Nanjing Jiancheng Bioengineering Institute (NJBI, Nanjing, China). Lipofectamine 2000 reagent was obtained from Invitrogen (Carlsbad, USA).

### Animals

Specific pathogen free (SPF) male Balb/c mice (18–20 g) were purchased from Beijing Huafukang Biotechnology Co., Ltd (Bejing, China). The mice were kept in specific pathogen free condition and an environmentally controlled room (23 ± 2°C, 55% ± 10% humidity) on a 12 h-light / dark cycle and fed by sterilized fodder and water adlibitum. During feeding and study, body weight and health status of mice were monitored in specific pathogen free condition every day. The mice were euthanized when they fulfilled one of the following human end point criteria: (1) more than 10% decline in body weight within one day, (2) abnormal behaviors (continuous tremble, not response to stimulus), (3) abnormal enlargement of abdomen, (4) heavy asthma, (5) loss of ability to ambulate. No animals died prior to the experimental endpoint or became severely ill and no adverse outcomes were seen in animals. Throughout the course of this study, all animals were sacrificed after sodium pentobarbital anaesthesia, and all efforts were made to minimize suffering. This study was carried out in strict accordance with the Tab of Animal Experimental Ethical Inspection of Laboratory Animal Centre, College of Basic Medical Sciences, Jilin University (China). This study was approved by the ethics committee of the College of Basic Medical Sciences, Jilin Universit (China), ID Number: 2014(18).

### The Acute Liver Injury Model of Mice Induced by CCl_4_

Mice were randomly divided into two groups. Acute liver injury of mice was induced by intraperitoneal injection of 0.5 ml CCl_4_ in olive oil (1:19 v/v)/kg body weight, and control mice were intraperitoneally injected with 10 ml of olive oil/kg body weight. Mice were executed on day 1, 3 and 5 after injection.

### Smad3-Overexpression *in vivo* in Mice

Mice were treated *in vivo* with Smad3-expressing plasmid pcDNA-Smad3 and control empty plasmid pcDNA3, respectively. Briefly, the pcDNA-Smad3 plasmids or control pcDNA3 plasmids were enfolded by Lipofectamine 2000 (1: 2) according to the manufacturer’s protocol, and then injected into mice (3μg plasmids in 100 μl 0.85% NaCl / mouse) via hydrodynamic tail vein injection as previously described [21]. 12 h post-injection of plasmids, the mice were intraperitoneally injected with CCl_4_, and executed on day 1, 3 and 5 after treatment with CCl_4_.

### Detection of Serum Transaminases ALT and AST

The serum transaminases ALT and AST level was determined by assay kit according to the manufacturer’s protocol (NJBI, China) as described previously [17].

### Histopathological Evaluation

The mouse liver was fixed with 4% paraformaldehyde at room temperature (RT) for 24 h, then embedded in paraffin, sliced into sections with a thickness of 4 μm, and pathological changes of liver sections were examined by hematoxylin and eosin (H&E) staining. The liver injury index (the injured area / total liver area x100%) was calculated for each section.

### RT-PCR

Total RNA was extracted from liver tissue using a Trizol reagent in accordance with the manufacturer’s instructions (Takara Biotechnology Co., Dalian, China). 1μg RNA was reverse-transcribed with specific antisense primer by using the one-step reverse-transcription polymerase chain reaction (RT-PCR) kit in accordance with the manufacturer’s instructions (Takara). PCR was performed by using following reaction conditions, 94°C for 30 s, 55°C for 20 s and 72°C for 40 s, in which all were for 35 cycles, the final extension step was at 72°C for 10 min. PCR products were separated by electrophoresis with 1.5% agarose, and stained with ethidium bromide. The specific bands were analyzed using IMAGEMASTER VDS (Pharmacia Biotech Company, Uppsala, Sweden). Primers were synthesized by Generay Biotechnology (Shanghai, China), and primer sequences are shown in Table 1.

**Table 1:** Primer sequences used in the study.

Target Gene	Forward primer (5′-3′)	Reverse primer (5′-3′)
***TGF-β1***	GATTGTTGCCATCAACGACC	GTGCAGGATGCATTGCTGAC
***TβRII***	CTCCATGGCTCTGGTACTCT	CCAGCACTCGGTCAAAGTCT
***Smad2***	ATGGCCGTCTTCAGGTTTCACA	ACTCTGTGGCTCAATTCCTGCT
***Smad3***	CCAGCACACAATAACTTGGA	AGACACACTGGAACAGCGGA
***Smad4***	GAGGTGGCCTGATCTACACA	TGATGCGCGATTACTTGGCG
***Smad6***	GGTGTACGACCAGGCTGTCA	GATGGAGTAACCCGGTGGCA
***Smad7***	GGCATTCCTCGGAAGTCAAG	GGCTCCAGAAGAAGTTGGGA
***GAPDH***	GATTGTTGCCATCAACGACC	GTGCAGGATGCATTGCTGAC

### Determination of TGF-β1, IL-1β and IL-6 Levels

Hepatic tissue homogenate and serum of mice were collected as described previously [17].The levels of TGF-β1 and inflammatory cytokines IL-1β and IL-6 were determined by ELISA kits according to the manufacturer’s protocol (BD Biosciences, San Diego, USA).

### Western Blotting

Liver tissues were homogenized and centrifuged at 12,000×g at 4°C for 30 min, and then the protein concentration was determined by the Pierce^®^ BCA Protein Assay (Rockford, USA). The hepatic tissue homogenate (30 μg protein) was separated by electrophoresis with 12% SDS-PAGE gel, and transferred onto a polyvinylidene difluoride membrane. The membrane was incubated in primary antibodies against Smad3 (1:1000), phospho-Smad3 (1:300), Bax (1:1000), Bcl-2 (1:200), cytochrome C (1:1000) and the cleaved caspase 3 (1:300) overnight, respectively, and then probed with a horseradish peroxidase-conjugated second antibodies. Finally, the labeled proteins were detected by chemiluminescence (ECLPlus; Amersham Pharmacia Biotech).

### Immunohistochemistry

The sections of liver tissue were incubated with 3% methanol-hydrogen peroxide to block endogenous peroxidase for 30 min at RT. Nonspecific reactivity was blocked by a pre-incubation of sections in 2% bovine serum albumin (BSA) for 30min. The sections were incubated in rabbit anti-mouse Smad3 antibody (ABclonal) overnight at 4°C, and then processed with biotinylated secondary antibodies for 10 min at RT, and further incubated in streptomycete-horseradish peroxidase for 10min at RT. Immunoreactive products were visualized in 0.03% H_2_O_2_ and 0.05% diaminobenzidine (DAB). The sections were dehydrated, cleared and mounted, and observed under a light microscope and photograph taken. In order to control staining, sections were incubated with normal rabbit IgG instead of anti-Smad3 antibody.

### MPO staining

There are high myeloperoxidase (MPO) activities in neutrophil, so MOP staining was performed in neutrophil with benzidine method. Briefly, the sections of liver tissue were fixed with 4% paraformaldehyde for 24 h at RT, and then incubated in 0.2 mg/ml benzidine and 0.02% H_2_O_2_ for 2 min. After washed under water for 2 minutes, the sections were incubated with Giemsa staining for 30s, and then observed under a light microscope and photograph taken.

### Immunofluorescence staining

The sections of liver tissue were blocked with 2% BSA for 30 minutes at RT, and then incubated with APC-conjugated anti-F4/80 antibody (eBioscience, San Diego, CA) at RT for 30 minutes. After washing in PBS for three times, Each sections was observed under a confocal laser scanning microscope (Olympus IX71).

### Flow Cytometry

Single cell suspensions from liver were incubated in PE-conjugated anti-mouse CD14, Percp-Cy5.5-conjugated anti-mouse CD11b, and PE-conjugated anti-mouse Ly-6G for 30min at RT, respectively. The labeled cells were analyzed by flow cytometry (BD Calibur). Data were collected and analyzed with Cell Quest software (BD Biosciences) to get the percentage of fluorescence cells.

### TUNEL Staining

The deparaffinized sections of liver tissues were stained by TUNEL staining according to the manufacturer’s instruction (Roche, Mannheim, Germany) as described previously [22]. Slides were observed under fluorescent microscope and images were captured with a digital camera (Olympus IX71, Center Valley, PA, USA). Apoptotic index (positive cells/total cells x100%) was calculated for each section [23].

### Statistical Analysis

Data are expressed as mean ± standard deviation (SD), and statistical evaluation was performed by statistical software SPSS 17.0. Differences of *P* < 0.05 were considered statistically significant.

## Results

### The Acute Liver Injury of Mice Induced by CCl_4_

**Fig 1:**
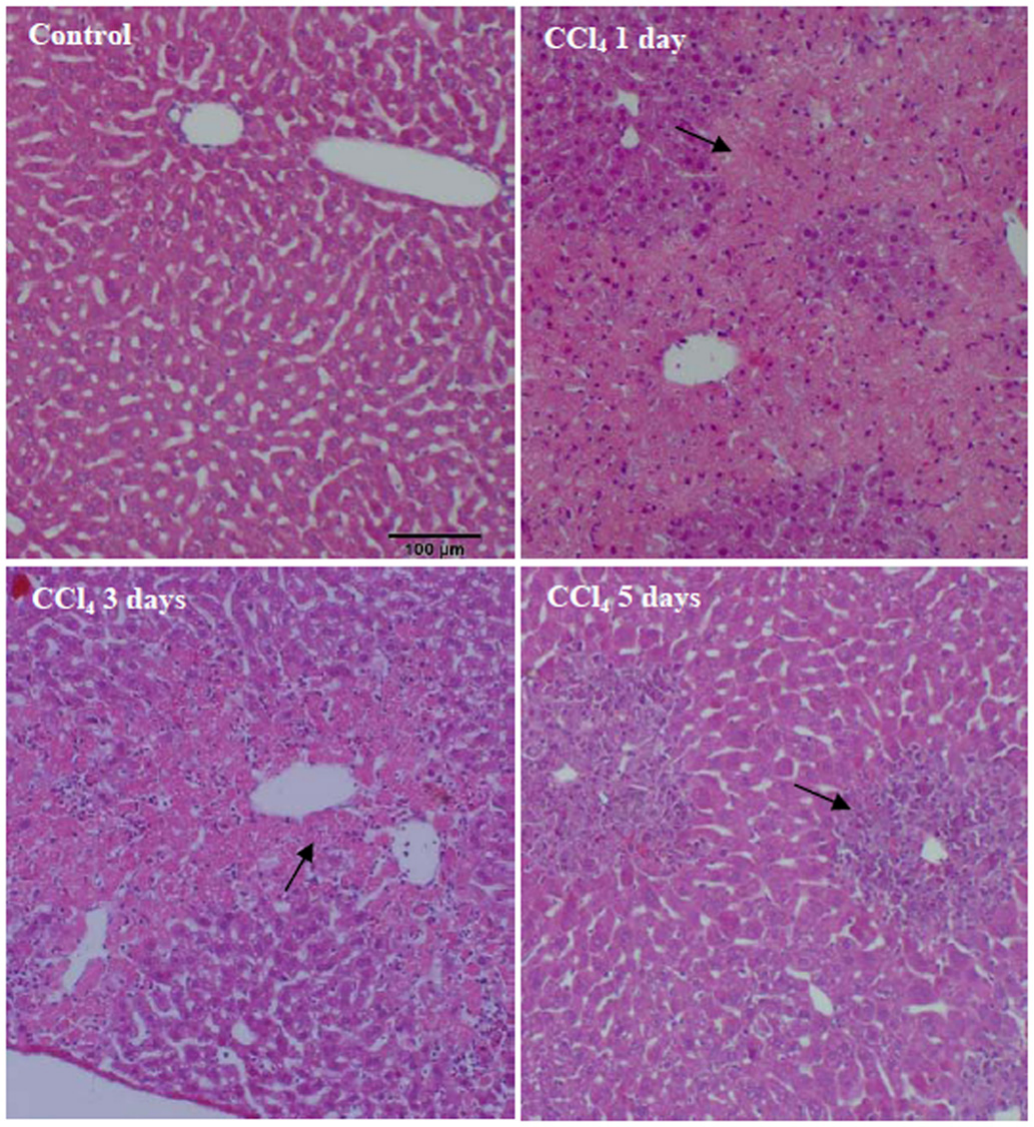
Pathological changes of livers in CCl_4_-treated mice. Pathological changes of livers were analyzed by H&E staining. Arrows represent lesion area.

In order to determine the damage severity, H&E staining was used to examine pathologic changes of mice livers. The results showed that lobular structure in the control mouse with olive oil treatment was clear and the hepatic cells arranged in neat rows. In livers of mice 1d following CCl_4_ treatment, there were large areas of annular necrotic lesions around the hepatic lobule portal area, and 3d after CCl_4_ treatment, there was more inflammatory cells infiltration in the hepatic lobule portal area. The injury liver repair and inflammatory cells infiltration could be observed 5d after CCl_4_ treatment (Fig 1). These data indicated that CCl_4_ successfully induced acute liver injury in mice.

### Levels of TGF-β1 in Sera of CCl_4_-treated Mice

**Fig 2:**
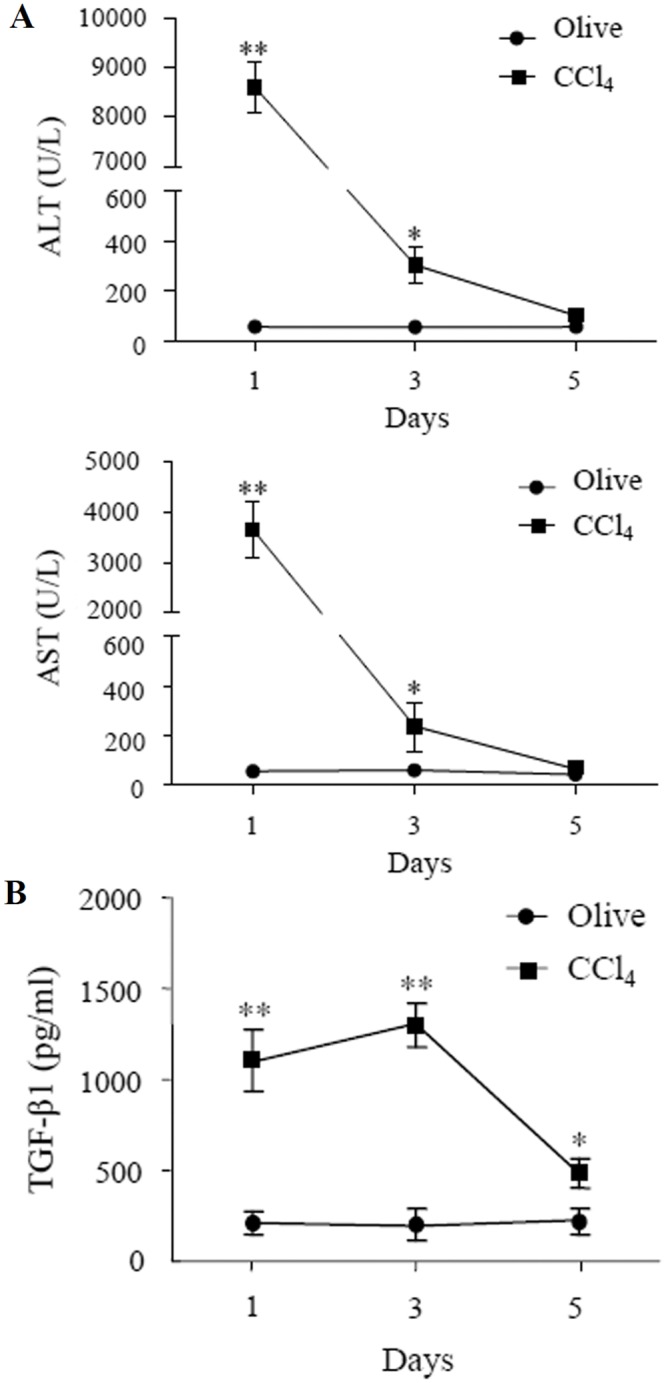
Levels of serum ALT and AST and TGF-β1 in mice treated with CCl_4_. (A) Levels of ALT and AST were detected in sera of mice treated with CCl_4_ (■) and control mice treated with olive oil (●). (B) Levels of TGF-β1 were determined by ELISA in sera of mice treated with CCl_4_ (■) and control mice treated with olive oil (●). *P<0.01, **P<0.001, compared with control group (n = 6).

Change of serum transaminases ALT and AST levels is an important indicator of liver injury. The results revealed that levels of ALT and AST in sera of mice were significantly elevated on days 1 and 3 after the administration of CCl_4_, compared with those in control mice treated with olive oil (Fig 2A), and the increased peak was on day 1, and then gradually declined. Simultaneously, the results revealed that TGF-β1 levels also increased significantly in sera of CCl_4_-treated mice, compared with those in control mice treated with olive oil (Fig 2B), and the levels peaked on day 1 and 3.

### Expression of TGF-β1 in Livers of CCl_4_-treated Mice

**Fig 3:**
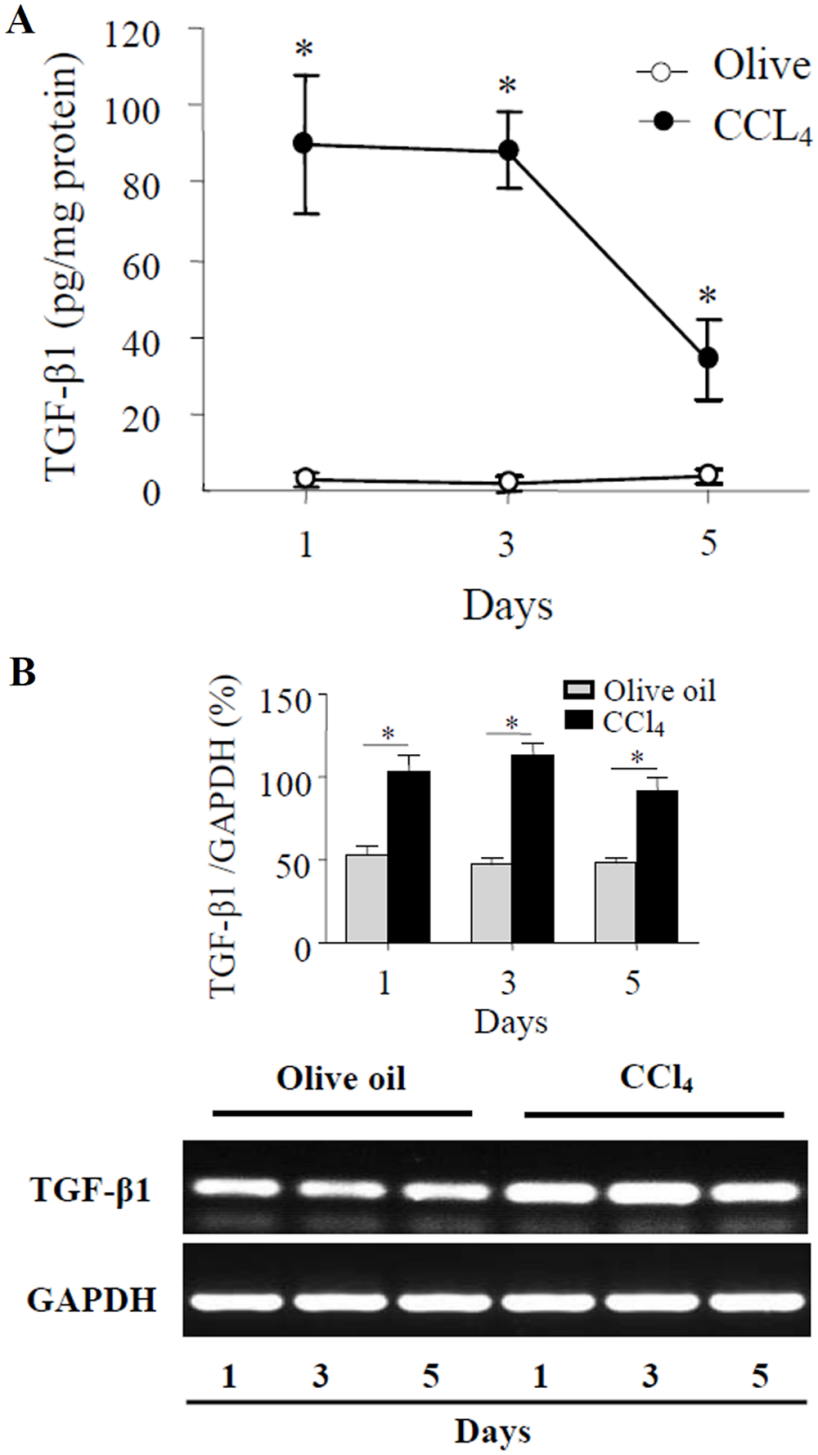
Expression of TGF-β1 in livers of mice treated with CCl_4_. (A) Levels of TGF-β1 were examined by ELISA in hepatic homogenates of mice with CCl_4_ (●) and control mice treated with olive oil (○). *P<0.01, compared with control group (n = 6). (B) Expression of *TGF-β1* mRNA was examined by RT-PCR in livers of mice treated with CCl_4_ and control mice treated with olive oil. The graph represents relative levels of *TGF-β1* mRNA from triplicate determinations. *P<0.01, compared with olive oil control group.

To further confirm TGF-β1 expression in the process of CCl_4_-induced acute liver injury in mice, TGF-β1 levels in hepatic tissue homogenate and *TGF-β1* mRNA expression in liver tissues were examined. The result showed that TGF-β1 protein levels increased significantly in hepatic tissue homogenates of CCl_4_-treated mice, compared with those in control mice treated with olive oil (Fig 3A), and the levels peaked on day 1 and 3, and then declined. RT-PCR results showed that *TGF-β1* mRNA expression was also up-regulated observably in livers of CCl_4_-treated mice (Fig 3B), compared with that in livers of control mice. These data above indicated that the levels of *TGF-β1* mRNA and protein increased obviously during CCl_4_-induced acute liver injury of mice.

### Expressions of TβRII and Smads in Livers of Mice Treated with CCl_4_

**Fig 4:**
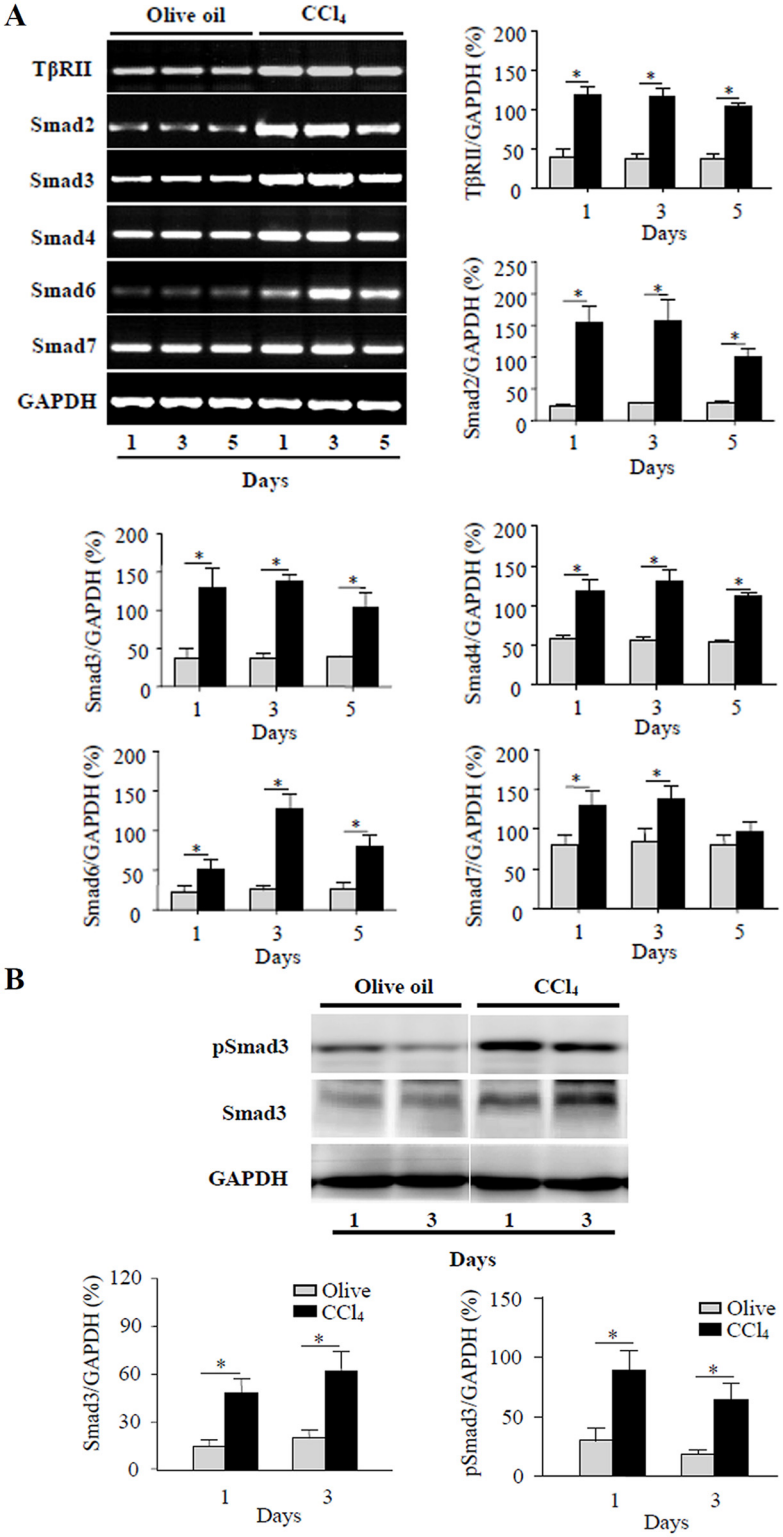
Expressions of TβRII and Smads mRNA and protein in livers of mice treated with CCl_4_. (A) Expressions of *TβRII* and signaling molecules *Smad2*, *3*, *4*, *6*, *7* mRNA were examined by RT-PCR in livers of mice treated with CCl_4_ and control mice treated with olive oil. The graph represents relative levels of mRNA from triplicate determinations. Olive oil (grey bars), CCl_4_ (black bars). *P<0.01, compared with olive oil control group. (B) pSmad3 and Smad3 protein levels were analyzed by Western blotting in livers of mice treated with CCl_4_ and control mice treated with olive oil. The graph represents relative protein levels of pSmad3 and Smad3 from triplicate determinations. *P<0.01, compared with olive oil control group.

Smad2 and Smad3 are engaged in signal transduction of TGF-β, whereas Smad1 and Smad5 are necessary for BMP signaling. To confirm the activation of TGF-β1/Smads signaling in CCl_4_-induced acute liver injury in mice, *TβRII* and *Smad2*, *3*, *4*, *6*, *7* mRNA expressions were examined by RT-PCR. The results showed that *TβRII* and *Smad2*, *3*, *4*, *6*, *7* mRNA expressions were dramatically augmented in livers of CCl_4_-treated mice, compared with those in livers of control mice (Fig 4A). Western blotting results revealed that Smad3 and phospho-Smad3 (pSmad3) protein levels were elevated significantly in CCl_4_-treated mice (Fig 4B), compared with those in control mice treated with olive oil. The above results revealed that TGF-β1/Smad3 signaling was activated in livers of CCl_4_-treated mice.

### Effects of Smad3 Overexpression on CCl_4_-induced Acute Liver Injury Severity

**Fig 5:**
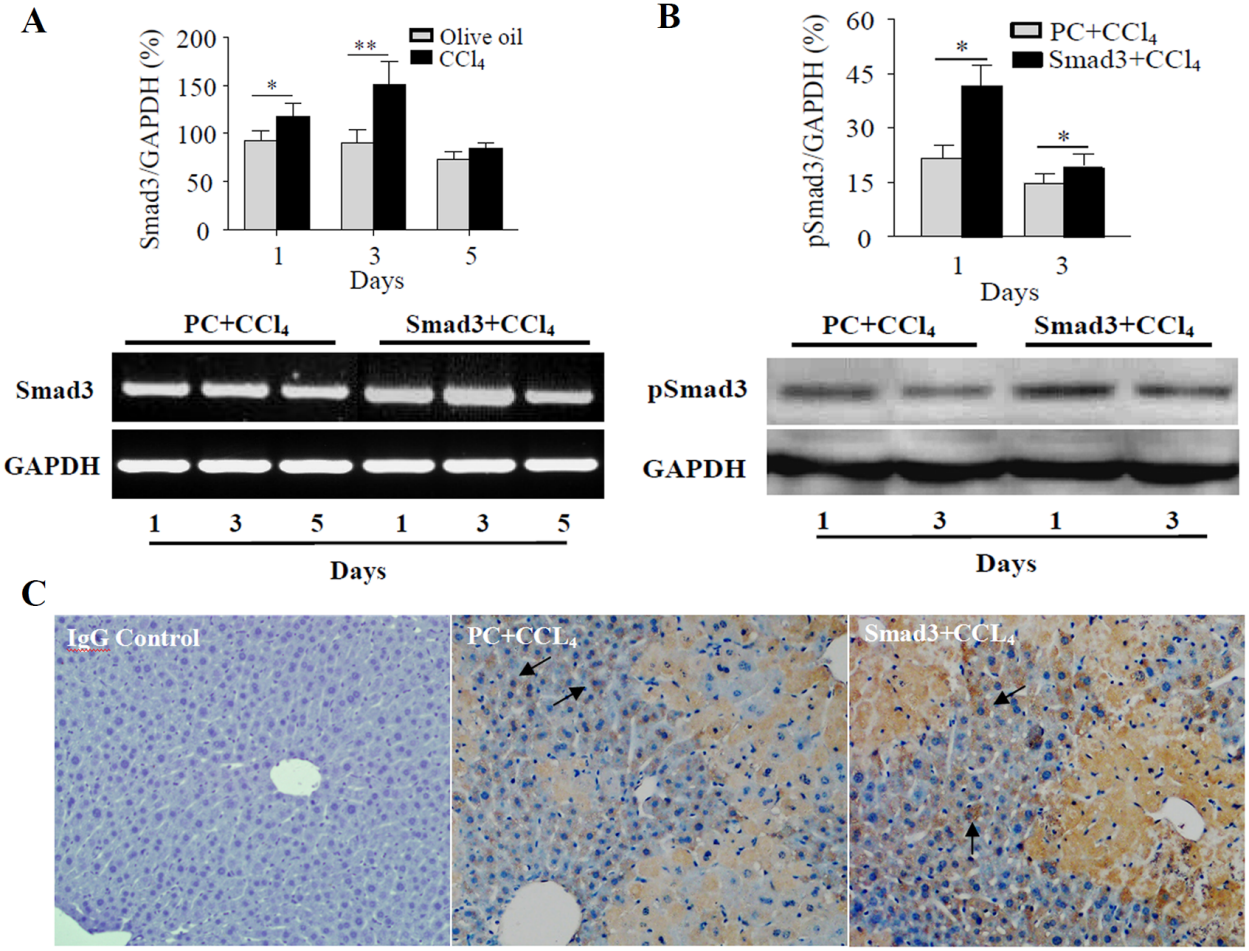
Expression of Smad3 in livers of Smad3-overexpressing mice. (A) *Smad3* mRNA expressions were evaluated by RT-PCR in livers of CCl_4_-treated Smad3-overexpressing mice (Smad3 + CCl_4_) and plasmid control mice (PC + CCl_4_). The graph represents relative levels of *Smad3* mRNA from triplicate determinations. *P<0.05, **P<0.01, compared with plasmid control group. (B) pSmad3 protein levels were examined by Western blotting in livers of CCl_4_-treated Smad3-overexpressing mice (Smad3 + CCl_4_) and plasmid control mice (PC + CCl_4_). The graph represents relative protein levels of pSmad3 from triplicate determinations. *P<0.01, compared with plasmid control group. (C) Expression of Smad3 protein was examined by immunohistochemical staining with rabbit anti-mouse Smad3 antibody in livers of Smad3-overexpressing mice (Smad3 + CCl_4_) and plasmid control mice (PC + CCl_4_) treated with CCl_4_ for 1 day. A procedural control staining in the liver was performed using normal rabbit IgG instead of anti-Smad3 antibody (Control). Arrows represent Smad3 positive cells (x100).

In order to determine the effect of the elevated TGF-β1/Smad3 signaling on CCl_4_-induced acute liver injury in mice, *in vivo* Smad3-overexpressing mice was prepared by tail intravenous injection with Smad3-expressing plasmid pcDNA-Smad3. The results showed apparently higher levels of *Smad3* mRNA and pSmad3 protein in liver of Smad3-overexpressing mice than those in the control plasmid mice upon treatment of CCl_4_ (Fig 5A and 5B), and expression of Smad3 protein was up-regulated in hepatocytes of Smad3-overexpressing mice confirmed by immunohistochemical staining (Fig 5C), indicating the successful establishment of Smad3-overexpression liver model of mouse.

**Fig 6:**
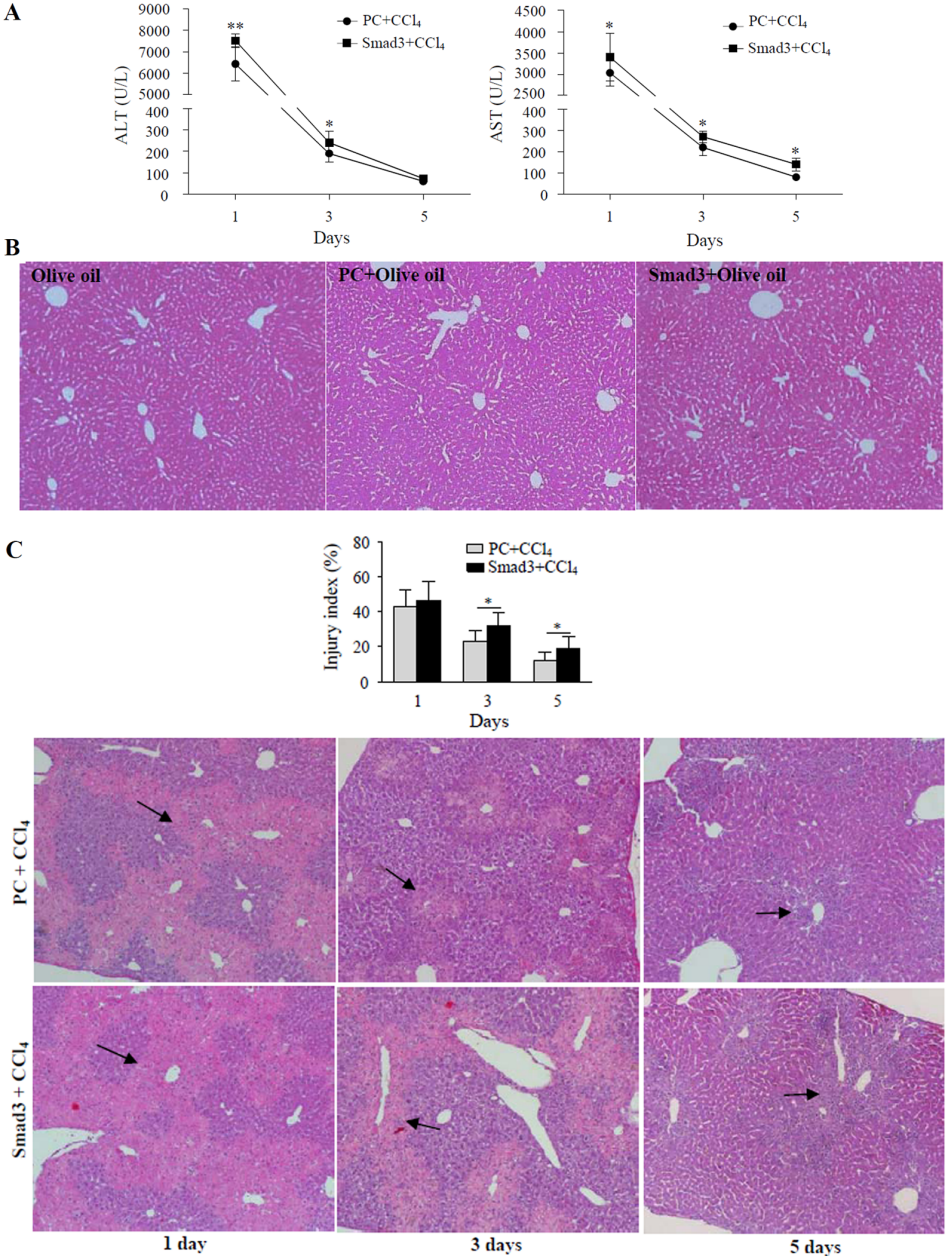
Effects of Smad3 overexpression *in vivo* on liver injury of CCl_4_-treated mice. (A) Levels of ALT and AST were examined by ELISA in sera of CCl_4_-treated Smad3-overexpressing mice (■) and plasmid control mice (●). *P<0.05, **P<0.01, compared with control group (n = 6). (B) Pathological changes of livers were analyzed by H&E staining in 1 day olive oil-treated control mice (Olive), plasmid control mice (PC + Olive) and Smad3-overexpressing mice (Smad3 + Olive) (x40). (C) Pathological changes of livers were analyzed by H&E staining in CCl_4_-treated Smad3-overexpressing mice (Smad3 + CCl_4_) and plasmid control mice (PC + CCl_4_). Arrows represent lesion area (x40). The graph represents liver injury index. *P<0.05.

Simultaneously, the results showed higher levels of serum ALT and AST in the Smad3-overexpressing mouse on day 1, 3, 5 after the administration of CCl_4_ compared with those in the control plasmid mouse treated with CCl_4_ (Fig 6A). H&E staining showed no liver injury in the Smad3-overexpressing mice and the plasmid control mice treated with olive oil for 1 day, compared with olive oil-treated control mice (Fig 6B). The results further revealed no significant difference in area of annular necrotic lesions around the hepatic lobule portal between the Smad3-overexpressing mice and the plasmid control mice on day 1 after CCl_4_ treatment. However, on day 3 and 5 after CCl_4_ treatment, larger area of annular necrotic lesions with massive inflammatory cells infiltration was observed in the Smad3-overexpressing mice, compared with the control mouse (Fig 6C). These data indicated that Smsd3 overexpression *in vivo* aggravated CCl_4_-induced acute liver injury in mice and prevented repair of the injured liver.

### Determination of Inflammatory Cells in Livers of Smad3-Overexpressing mice

**Fig 7:**
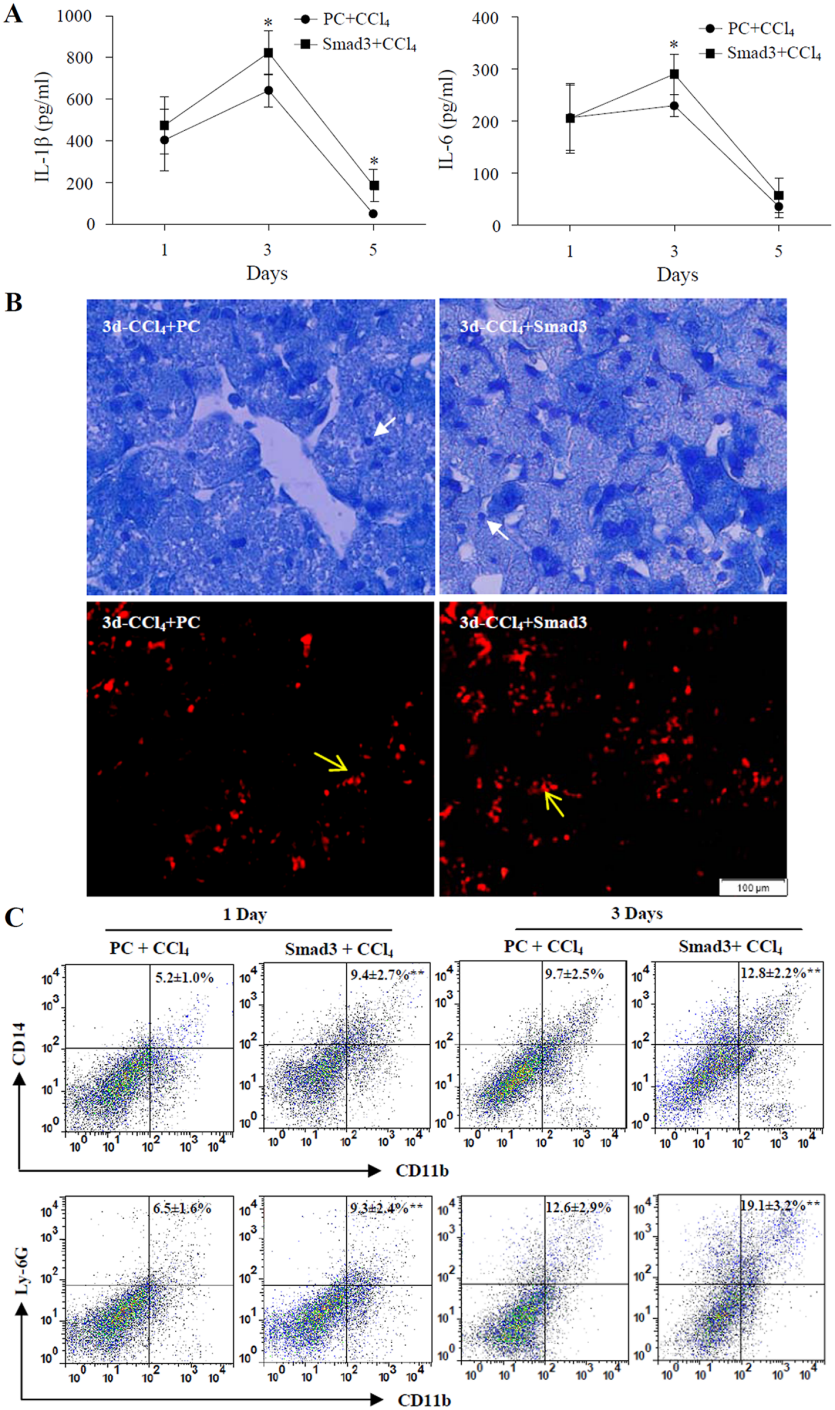
Assay of inflammatory cytokines and inflammatory cells in Smad3-overexpressing mice. (A) Levels of IL-1β and IL-6 were examined by ELISA in sera of CCl_4_-treated Smad3-overexpressing mice (■) and plasmid control mice (●). *P<0.05, compared with control group (n = 6). (B) The infiltration of neutrophils and macrophages in livers of plasmid control mice (PC + CCl_4_) and Smad3-overexpressing mice (Smad3 + CCl_4_) treated with CCl_4_ for 3 days were examined by MPO staining and F4/80 immunofluorescence staining. Arrows represent neutrophils (dark blue cells) (x400) and macrophages cells (red cells) (x200). (C) The inflammatory cells were examined in livers of CCl_4_-treated plasmid control mice (PC + CCl_4_) and Smad3-overexpressing mice (Smad3 + CCl_4_) by flow cytometry with anti-CD14/CD11b and anti-Ly-6G/CD11b antibodies. *P<0.05, **P<0.01, compared with control group (n = 6).

To further elucidate the roles of Smad3 overexpression in acute liver injury induced by CCl_4_, levels of inflammatory cytokines IL-1β and IL-6 in sera of mice were first determined by ELISA. The results revealed that IL-1β and IL-6 levels were increased apparently in sera of Smad3-overexpressing mice treated with CCl_4_, compared with those in plasmid control mice treated with CCl_4_ (Fig 7A). Next, the infiltration of inflammatory cells in livers of Smad3-overexpressing mice was examined by MPO staining and anti-F4/80 immunofluorescence staining. The results showed that MPO positive cells (neutrophils) and F4/80 positive cells (macrophages) increased in livers of Smad3-overexpressing mice treated with CCl_4_, compared with those in plasmid control mice treated with CCl_4_ (Fig 7B). Finally, the amount of inflammatory cells in livers were confirmed by flow cytometry. The results showed that the amount of macrophages (CD14^+^CD11b^+^) and neutrophils (CD11b^+^Ly-6G^+^) in livers of Smad3-overexpressing mice treated with CCl_4_ were remarkably higher than those in plasmid control mice treated with CCl_4_ (Fig 7C). These data indicated that Smad3 overexpression *in vivo* might promote the infiltration of inflammatory cells and release of inflammatory cytokines in mouse liver upon treatment of CCl_4_.

### Effects of Smad3 Overexpression on Hepatocytes Apoptosis in CCl_4_- treated Mice

**Fig 8:**
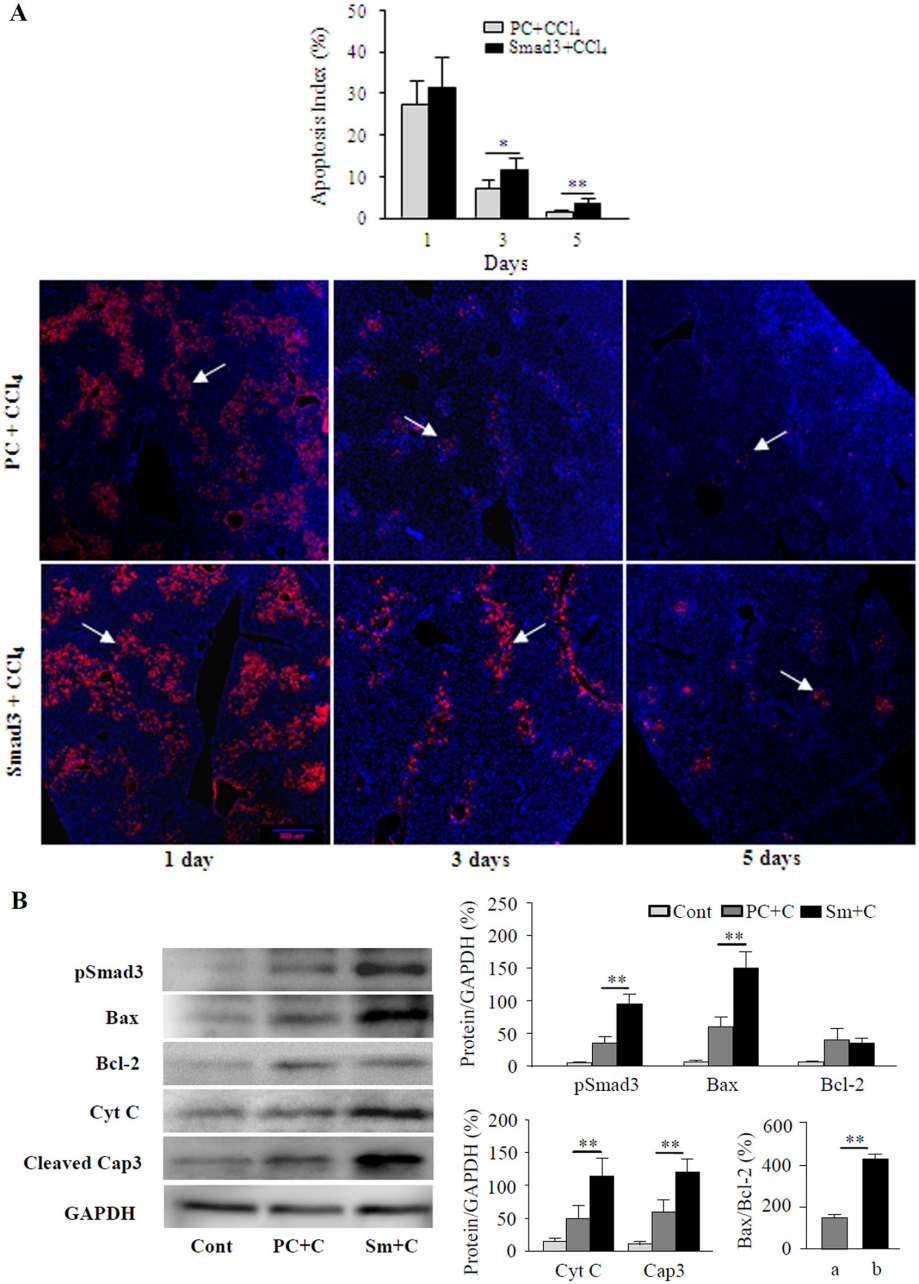
Analysis of hepatocytes apoptosis in Smad3-overexpressing mice. (A) The apoptosis of hepatocytes was examined by TUNEL staining in livers of CCl_4_-treated Smad3-overexpressing mice (Smad3 + CCl_4_) and plasmid control mice (PC + CCl_4_). Arrows represent apoptotic cells (red cells). The graph represents the apoptotic index of hepatocytes. *P<0.05 and **P<0.01. (B) Levels of pSmad3 and apoptosis-associated proteins Bax, Bcl2, Cyt C and the cleaved caspase 3 (cleaved cap3) were evaluated by Western blotting in livers of non-treated mice (Cont), CCl_4_-treated plasmid control mice (PC + C) and CCl_4_-treated Smad3-overexpressing mice (Sm + C). The graph represents levels of relative protein from triplicate determinations.*P<0.01. CCl_4_-treated Smad3-overexpressing mice (b) vs CCl_4_-treated plasmid control mice (a).

To further explore the possible mechanism of roles of Smad3 overexpression in liver injury induced by CCl_4_, TUNEL staining was used to examine apoptosis of hepatocytes. The results showed that a large number of TUNEL-positive hepatocytes appeared in the hepatic lobule portal area of mice on day 1 and day 3 after the administration of CCl_4_ (Fig 8A), and significantly more TUNEL-positive hepatocytes were observed in the Smad3-overexpressing mice day 3 and day 5 after CCl_4_ treatment, compared with those in the plasmid control mice treated with CCl_4_.

Furthermore, western blotting analyses revealed that levels of not only pSmad3 protein, but also apoptosis-associated proteins Bax, Cyt C and the cleaved caspase 3 proteins were obviously elevated in livers of Smad3-overexpressing mice treated with CCl_4_, and Bax/Bcl-2 ratio was increased significantly on day 1 after the administration of CCl_4_ (Fig 8B). These above data demonstrated that Smad3 overexpression might aggravate CCl_4_-induced acute liver injury in mice by promoting hepatocytes apoptosis.

## Discussion

TGF-β family mainly consists of three members TGF-β1, TGF-β2 and TGF-β3, among which TGF-β1 is the most abundant and potent in tissues. TGF-β1 plays important roles in the process of genesis and development of many diseases. For example, it can prevent Epstein-Barr *virus* reactivation through modulating *H*. *pylori* infection [24]. and also be the important factor for inducing liver fibrosis, pulmonary fibrosis and chronic nephrosis [20, 25]. TGF-β signaling pathway was mediated by the serine/threonine kinase receptor on the cell surface and intracellular Smad proteins [26]. The activated TGF-β receptors propagate the signal through phosphorylation of the Smad proteins. Smad 1, Smad 5 and Smad 8 are activated in response to BMP signals, Smad 2 and Smad 3 participate in TGF-β signals, and Smad 4 acts as a common mediator of TGF-β and BMP signals [27].

A large number of studies have shown that TGF-β1/Smad3 signaling participates in the genesis and development of various diseases. TGF-β1/Smad3 signaling can stimulate human ovarian cancer cell migration by up-regulating connexin43 expression [28],regulate insulin gene transcription and pancreatic islet cell function via Smad3 signaling [29].and play a critical role in renal fibrosis and chronic liver disease [30, 31]. However, the effect of TGF-β1/Smad3 signaling on the CCl_4_-induced acute liver injury is still unclear. In this study, we established the CCl_4_-induced acute liver injury model of mice, and found that not only the levels of liver injury markers ALT and AST in serum, but also TGF-β1 levels in serum and hepatic tissue homogenate increased significantly in mice treated with CCl_4_. Furthermore, the mRNA expressions of *TβRII*, *Smad2 and Smad3* were greatly up-regulated in liver tissues of mice treated with CCl_4_, and similar increase of phosphorylated and non-phosphorylated Smad3 proteins was observed. These data indicated that TGF-β1/Smad3 signaling pathway was activated and might be involved in the process of CCl_4_-induced acute liver injury.

Previous studies have reported that overexpression of Smad3 is closely linked with a variety of diseases. For example, Smad3 can promote renal fibrosis and chronic liver disease [30, 31].Otherwise, the increased *Smad3* mRNA expression has been reported in CCl_4_-induced acute liver injury mice [17]. In the present study, we examined the effect of Smad3 overexpression in the mouse liver on CCl_4_-induced acute liver injury by tail vein injection of the Smad3-expressing plasmid. ALT and AST levels in serum were significantly elevated in CCl_4_-treated Smad3-overexpressing mice, compared with those in CCl_4_-treated control mice. H&E staining results further showed that liver tissues of Smad3-overexpressing mice treated with CCl_4_ had larger areas of annular necrotic lesions with massive inflammatory cells infiltration than those in control mice treated with CCl_4_. These results indicated that overexpression of Smad3 might aggravate CCl_4_-induced acute liver injury.

Macrophages, neutrophils, nature killer (NK) cells and NKT cells have been shown to exist in liver tissue [32]. Macrophages, lymphocytes and neutrophils play an important role in secreting cytokines during liver injury [33, 34]. The previous study has reported that Smad3 can induce the production of IL-6 in liver tissue [35]. Our data revealed that Smad3 overexpression could promote the infiltration of inflammatory cells in liver, such as macrophages and neutrophils in liver of Smad3-overexpressing mice treated with CCl_4_, concomitant with an increase in the serum level of IL-1β and IL-6. These data suggested that Smsd3 overexpression promoted the infiltration of inflammatory cells and induced the release of inflammatory cytokines, which might aggravate CCl_4_-induced acute liver injury and prevented repair of the injured liver.

Previous studies have shown that TGF-β1 can inhibit hepatocellular DNA synthesis and cell proliferation [36, 37]. In this study, a large number of apoptotic hepatocytes was observed in CCl_4_-induced acute liver injury model mice, and the apoptosis of hepatocytes were obviously augmented in Smad3-overexpressing mice treated with CCl_4_. Additionally, *in vitro* Smad3 overexpression slight increased apoptosis of mouse hepatoma cell line Hepa1-6 cells treated with CCl_4_, but there was no significant difference, compared with apoptosis of the plasmid control cells treated with CCl_4_ (shown in S1 Fig). This result is similar to that there was no significant difference of hepatocytes apoptosis between *in vivo* Smad3-overexpressing mice and plasmid control mice day 1 after CCl_4_ treatment. These data suggest that Smad3 overexpression can not promote hepatocytes apoptosis induced by CCl_4_ directly, while it may mainly mediate TGF-β1-induced secondary hepatocytes apoptosis to prevent the liver injury repair. Consistently, our results revealed that Smad3 overexpression promoted the expression of apoptosis-related proteins Bax, Cyt C and caspase 3, and resulted in a higher ratio of Bax/Bcl-2. Apoptosis can occur through three major pathways, including the ER stress, the mitochondrial and the Fas death receptor pathways [38–40]. Bax, Bcl-2 and Cyt C are mainly involved in the mitochondrial pathway [40, 41]. Therefore, Smad3 overexpression *in vivo* might aggravate CCl_4_-induced acute liver injury in mice by inducing hepatocytes apoptosis through the mitochondrial pathway.

In conclusion, the activation of TGF-β1/Smad3 signaling in mice of CCl_4_-induced acute liver injury might aggravate liver injury severity and prevent repair of the injured liver by promoting inflammatory cells infiltration, inflammatory cytokines release, and apoptosis of hepatocytes through the mitochondrial pathway. Therefore, these results support the TGF-β1/Smad3 signaling as an important target for acute liver injury therapy.

## Supporting Information

S1 Fig Effect of Smad3 overexpression on apoptosis of Hepal-6 cells. (PDF)
