# Supplementary material for: Involvement of TGF-β1/Smad3 Signaling in Carbon Tetrachloride-Induced Acute Liver Injury in Mice
Source: PLoS One. 2016 May 25;11(5):e0156090. doi: 10.1371/journal.pone.0156090 (PMC4880333; doi:10.1371/journal.pone.0156090)
Supplement: S1 Fig — (PDF) [file pone.0156090.s001.pdf]

Supporting information

Methods

Cell Culture

Mouse hepatoma cell line Hepal-6 cells were cultured in DMEM medium (GIBCO Life Technologies) supplemented with 10% FCS in a humidified atmosphere with 5% CO_2 at 37 ℃ incubator.

Transfection of Smad3-expressing Plasmid

Hepal-6 cells were seeded into 12 well culture plates at a density of $5x10^5$ cells/well, and transfected with Smad3-expressing plasmid pcDNA-Smad3 and control pcDNA3 plasmids, respectively. 12 hours after the transfection, 10mmol/L CCl_4 was added to per well. 12h later, Hepal-6 cells apoptosis was examined by flow Cytometry.

Flow Cytometry for Apoptosis Analysis

Hepal-6 cells were washed in cold PBS, collected in 100 μl binding buffer and stained with both 2μl propidium iodide (PI) and annexin V-FITC for 10 minutes according to the manufacturer's instruction (Roche, Mannheim, Germany). Data from 10,000 cells/sample were collected, and the data were analyzed with Cell Quest software (BD Biosciences) to assess the percentage of fluorescence positive cells.

Results

Effects of Smad3 Overexpression on Hepal-6 Cells Apoptosis

Hepal-6 cells were transfected with Smad3-expressing plasmid and control plasmid, respectively, and then Hepal-6 cells apoptosis was

examined by flow cytometry. The ratio of Hepal-6 cells apoptosis in Smad3-overexpressing Hepal-6 cells (Smad3 + CCl_4) is slight higher than plasmid control Hepal-6 cells (PC + CCl_4), but there was no statistically significant difference (S1 Fig). These data indicated that Smad3 overexpression *in vitro* might not promote the hepatocytes apoptosis induced by CCl_4 directly.

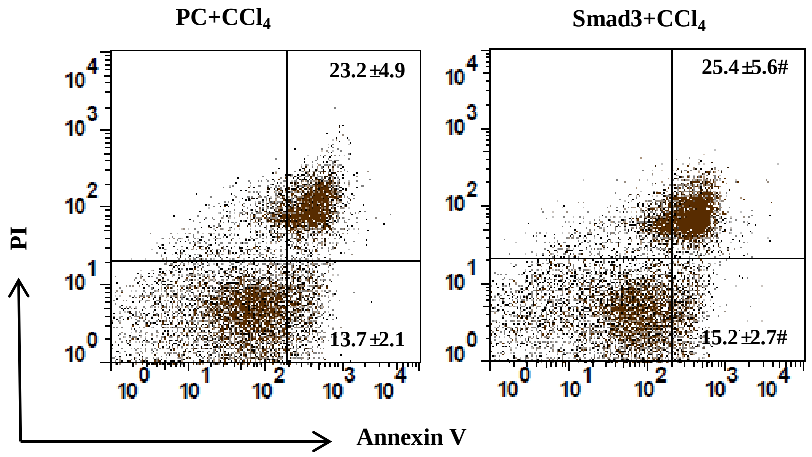

S1 Fig. Effect of Smad3 overexpression on apoptosis of Hepal-6 cells. The cells apoptosis was examined by flow cytometry with PI and annexin V-FITC staining in Smad3-overexpressing Hepal-6 cells (Smad3+CCl_4) and plasmid control Hepal-6 cells (PC+CCl_4) treated with CCl_4. A representative experiment of the three performed is shown. AnnexinV+/PI- represents early apoptosis, and AnnexinV+/PI+ represents late apoptosis. #*P*>0.05, compared with plasmid control group.
